# A novel de novo androgen receptor nonsense mutation in a sex-reversed 46,XY infant

**DOI:** 10.1038/s41439-021-00167-5

**Published:** 2021-09-01

**Authors:** Kok-Siong Poon, Karen Mei-Ling Tan, Kah Yin Loke

**Affiliations:** 1 Department of Laboratory Medicine, National University Hospital, Singapore, Singapore; 2 Division of Paediatric Endocrinology, Department of Paediatrics, Khoo Teck Puat - National University Children’s Medical Institute, National University Hospital, Singapore, Singapore

**Keywords:** Genetic testing, Hypogonadism

## Abstract

An infant with 46,XY karyotype, and unambiguous female phenotype was found to have testes in the inguinal regions. Capillary sequencing of the androgen receptor (*AR*) gene identified a hemizygous de novo mutation (NM_000044.6:c.1621G > T) in exon 2 resulting in a termination codon p.(Glu541*) at the DNA binding domain (DBD). This novel nonsense mutation adds to the compendium of *AR* mutations which result in complete androgen insensitivity syndrome (AIS).

Androgens play a role in sex differentiation and the development of male sexual characteristics. Their action is mediated by the AR, a nuclear steroid transcription factor. The *AR* gene is located on chromosome Xq11-12 and codes for the AR, which consists of 919 amino acids in length. The N-terminal domain NTD (amino acids 1–537) is encoded by exon 1 and is the largest domain in the AR, comprising more than half of the receptor, while the DBD is encoded by exons 2–3 and the ligand binding domain (LBD) by exons 4–8^1^. Pathogenic variants in the *AR* gene cause AIS (OMIM 300068) characterized by complete or partial resistance to the biological actions of androgens in an individual with XY karyotype with normal testes and production of age-appropriate androgen concentrations. The prevalence of AIS is estimated to range from 1/20,400 to 1/99,100 males^2^. The majority of pathogenic alterations are missense variants causing dysfunctional LBD and DBD^2,3^. Recent case reports described nucleotide changes found in deep intronic regions which activate cryptic splice sites and expression of aberrant *AR* transcripts in AIS patients^4–6^.

The phenotype of AIS includes (1) normal female external genitalia (complete AIS (CAIS)), (2) ambiguous genitalia or male with hypospadias and micropenis (partial AIS (PAIS)), and (3) normal male phenotype with infertility (mild AIS (MAIS)) in 46,XY individuals. CAIS often presents in infancy as an inguinal hernia or labial swelling containing a testis in an apparently normal female or may present as a mismatch between prenatal sex prediction (XY) and phenotype at birth (female)^2^. Patients with CAIS do not have a cervix or uterus and have a blind-ending vagina. Serum testosterone concentrations are within or above the normal range for boys, while luteinizing hormone and anti-mullerian hormone concentrations are increased^2^. The diagnosis of CAIS is usually based on clinical presentation and laboratory findings. Determination of testosterone (T), androstenedione (A), and dihydrotestosterone (DHT) concentrations are required to exclude testosterone biosynthesis defects or 5-alpha reductase deficiency^7^. Management of AIS, in particular CAIS, requires a multidisciplinary team including an endocrinologist, urologist, gynecologist, and psychologist to address functional, sexual, and psychological issues^2^. For CAIS presenting in infancy, gonadectomy may be performed with puberty induction, as there is a potential risk of gonadal tumor^2^.

We report a 1-month-old female infant who was born full term of normal vaginal delivery, with a birth weight of 2.54 kg. She was discharged well on day 3 of life but was noted to have a bilateral inguinal hernia at 1 week of life. On examination, there were reducible inguinal hernias with normal unambiguous female external genitalia, for which she was admitted for a bilateral herniotomy. At surgery, a biopsy was taken from the mass resembling a testis seen in the left inguinal hernia. Further exploration demonstrated the presence of testes in both inguinal regions in the absence of a uterus, which was subsequently demonstrated with pelvic and inguinal ultrasound. Histopathology of the biopsy showed seminiferous tubules.

This is the first child of non-consanguineous parents, with no family history of any disorder of sexual development (DSD) or AIS. Chromosomal karyotyping by G-banding revealed a male karyotype, 46,XY. The human chorionic gonadotropin stimulation test demonstrated a significant rise in testosterone concentration from 0.93 to 19.75 mmol/L. The child also had a normal testosterone (T): dihydrotestosterone (DHT) ratio of 8.9 (reference <20) and a normal testosterone (T): androstenedione (A) ratio of 2.8 (reference >0.8). A preliminary diagnosis of CAIS was made.

Capillary sequencing of the *AR* gene was performed with informed consent. A hemizygous variant NM_000044.6:c.1621G > T, NP_000035.2:p.(Glu541*) was identified (Fig. 1) in the infant’s blood DNA but not in the mother. Quantitative pyrosequencing showed 100% variant (T) allele in the proband and 100% normal (G) allele in the mother (Fig. 1). These results suggest that it was a de novo mutation in the proband, and somatic mosaicism is unlikely in the proband and the mother. A list of polymerase chain reaction, capillary sequencing, and pyrosequencing primers for *AR* exon 2 mutational analysis was available in Supplementary Table 1.

**Fig. 1:**
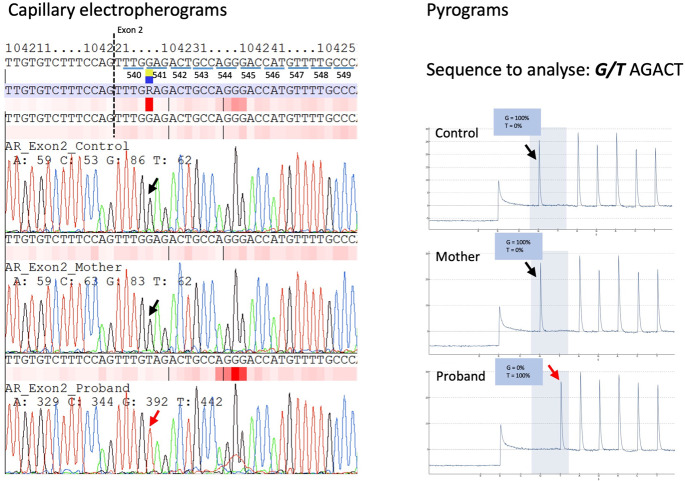
Detection of novel *AR* variant by capillary sequencing and pyrosequencing. Capillary electropherograms (left) and pyrograms (right) show the region encompassing the variant (NM_000044.6:c.1621G > T) in the normal control (top), mother (middle), and proband (bottom). Left: The capillary electropherograms were aligned against *AR* RefSeq NG_009014.2 and annotated. Hemizygous mutant (G > T) nucleotide was identified in proband’s blood DNA (indicated by red arrow). Right: 100% mutant (T) allelic fraction was quantified by pyrosequencing in the proband’s blood DNA (indicated by red arrow).

To our knowledge, this variant has not been previously described in the literature and is not present in the AR database (http://androgendb.mcgill.ca/), ClinVar (http://www.clinvar.com), Human Gene Mutation Database Professional 2020.4, and GenomAD (https://gnomad.broadinstitute.org/). The nonsense variant in exon 2 of the *AR* gene introduces a termination codon, which is expected to abolish the DBD and beyond. This variant is classified as pathogenic in the context of X-linked recessive AIS (PVS1, PM2, and PP4) according to the ACMG/AMP guidelines for the interpretation of sequence variants^8^. Thus the molecular investigation confirmed the diagnosis of CAIS.

Since 30% of AR mutations in AIS occur de novo, sequencing of the *AR* gene is recommended for patients with 46, XY DSD, regardless of family history^5,6^. Somatic mutations in the *AR* gene have also been rarely reported^3,9,10^. Our case illustrates that the presence of an inguinal hernia in a normal unambiguously female infant is a clear indication to screen for CAIS. The limitation in our molecular investigation was that only one tissue type, the blood, was tested. Pyrosequencing is a sensitive platform which has a 5% limit of detection of the minor allele fraction. The absence of the proband’s mutation in the mother’s blood DNA does not therefore completely reassure the family with the absence of recurrent risk of AIS in their future offspring, and future pregnancies should be carefully monitored although the risk is nevertheless low based on the current findings.

Since the diagnosis was CAIS, the parents decided to raise the child as a girl, with testes removal before puberty. Bilateral orchidectomy was performed when the child was one and a half years of age. During the laparoscopic examination, there was no uterus and there was a blind-ending vagina. Histopathology showed immature testicular tubules with no spermatogonia and no malignancy. In conclusion, we identified a novel de novo nonsense mutation in the *AR* gene in a patient with CAIS presenting with bilateral inguinal hernia.

## HGV Database

The relevant data from this Data Report are hosted at the Human Genome Variation Database at 10.6084/m9.figshare.hgv.3084.

## Supplementary information

Supplementary Table 1
