# Supplementary material for: A novel de novo androgen receptor nonsense mutation in a sex-reversed 46,XY infant
Source: Hum Genome Var. 2021 Sep 1;8:35. doi: 10.1038/s41439-021-00167-5 (PMC8410801; doi:10.1038/s41439-021-00167-5)
Supplement: Supplementary file 1 — Supplementary Table 1 [file 41439_2021_167_MOESM1_ESM.docx]

**Supplementary Table 1** List of polymerase chain reaction (PCR), capillary sequencing and pyrosequencing primers for *AR* exon 2 mutational analysis. NA: Not Applicable.

| Assay | Reaction | Oligonucleotide 5’ – 3’ | | PCR product size (base pairs) | Genomic coordinates (GRCh38 chrX) |
| --- | --- | --- | --- | --- | --- |
|  |  | Forward primer | Reverse primer |  |  |
| Capillary sequencing | PCR amplification | GCCTGCAGGTTAATGCTGAAGACC | CCTAAGTTATTTGATAGGGCCTTGCC | 379 | 67643164 .. 67643542 |
|  | Cycle sequencing | Same as the above | Same as the above | NA | NA |
| Pyrosequencing | PCR amplification | TTGCCTATTTCTGCCATTCA | GAAGACCTTGCAGCTTCCAC | 190 | 67643199 .. 67643388 |
|  | Sequencing | GTTTTTTGTGTCTTTCCAGTTT | NA | NA | NA |
